# Supplementary material for: Limitation of Futile Therapy in the Opinion of Nursing Staff Employed in Polish Hospitals—Results of a Cross-Sectional Study
Source: Int J Environ Res Public Health. 2022 Dec 17;19(24):16975. doi: 10.3390/ijerph192416975 (PMC9778965; doi:10.3390/ijerph192416975)
Supplement: Supplementary file 1 [file ijerph-19-16975-s001.zip › ijerph-2029903-supplementary.pdf]

Supplementary Table S1. Declarations of the respondents concerning the person that should decide about limiting futile therapy.

Variable	In adults						In children					
	yes		no opinion		no		yes		no opinion		no	
	n	%	n	%	n	%	n	%	n	%	n	%
Patient in a declaration of will/living will	160	84.21	21	11.05	9	4.74	-	-	-	-	-	-
Doctor	122	64.21	41	21.58	27	14.21	123	64.74	35	18.42	32	16.84
Head of the ward	89	46.84	55	28.95	46	24.21	82	43.16	55	28.95	53	27.89
Patient's family	94	49.47	46	24.21	50	26.31	58	30.53	63	33.16	67	35.26
Court	53	27.89	63	33.16	70	36.84	122	64.21	31	16.31	37	19.47

n – number, % – percentage

Supplementary Table S2. Differentiation of the percentage of answers on futile therapy in the groups defined by the qualitative variables: gender, place of employment, education, and religiousness (p values that are statistically significant or borderline significant are in bold).

Questionnaire question/ % of affirmative responses	Gender			Work - Children's Ward			Work - ICU			Education			Believer		
	F	M	p	Yes	No	p	Yes	No	p	L	H	p	Yes	No	p
Sufficient preparation to talk about futile therapy	6.95	16.67	0.21	5.13	5.56	**0.05**	8.33	4.69	**0.03**	10.00	5.84	0.68	6.94	5.88	0.84
The use of futile therapy is a mistake	31.61	33.33	0.10	43.59	23.33	**0.01**	40.00	17.19	**0.003**	30.00	33.12	0.61	31.79	29.41	0.95
Idea of limiting futile therapy is right	64.94	66.67	0.94	67.53	58.89	0.19	71.67	55.56	**0.001**	53.33	69.28	0.13	65.12	70.59	0.50
Comfort of the patient's last days of life is more important than futile therapy	90.32	94.44	0.56	92.31	87.78	0.33	96.67	81.25	**0.0004**	90.00	90.91	0.83	90.75	94.12	0.64
Permission to limit futile therapy in relation to oneself	75.66	62.5	0.20	80.52	66.29	0.11	79.83	77.78	0.55	62.07	76.67	0.19	73.37	81.25	0.56

		Gender			Work - Children's Ward			Work - ICU			Education			Believer		
		F	M	p	Yes	No	p	Yes	No	p	L	H	p	Yes	No	p
Decision to limit futile therapy for adults should be made by	patient	83.23	94.44	0.43	85.90	80.00	0.26	74.17	46.88	**0.001**	86.67	83.12	0.38	83.24	94.12	0.45
	doctor	67.10	50.00	0.31	74.36	53.33	**0.01**	59.17	25.00	**0.0003**	43.33	68.18	**0.03**	64.74	58.82	0.70
	head of the ward	49.03	38.89	0.71	52.56	38.89	0.19	86.67	79.69	0.11	40.00	48.05	0.17	47.40	41.18	0.49
	patient's family	50.32	55.56	0.38	55.13	46.67	0.39	52.50	45.31	0.50	56.67	48.70	0.65	51.45	29.41	0.14
	court	28.48	38.89	0.64	30.77	23.86	0.56	31.90	20.31	0.23	17.24	31.13	0.23	28.99	23.53	0.70
Decision on limiting futile therapy for children should be made by	doctor	65.81	61.11	0.91	74.36	52.22	**0.01**	73.33	50.00	**0.006**	46.67	68.18	0.07	64.74	64.71	0.75
	head of the ward	43.87	50.00	0.77	52.56	31.11	**0.01**	57.50	18.75	**<0.0001**	40.00	43.51	0.86	43.35	41.18	0.43
	patient's family	29.41	44.44	0.42	39.74	21.35	**0.03**	33.05	25.00	0.16	13.79	35.29	0.06	31.58	23.53	0.77
	court	65.81	66.67	0.99	66.67	62.22	0.61	60.00	71.88	0.15	76.67	61.69	0.25	65.90	47.06	0.22
Who would decide to limit futile therapy in relation to the subject	declaration of will	88.89	94.44	0.75	88.46	86.52	0.21	88.24	87.30	0.51	89.66	88.24	0.85	88.30	88.24	0.89
	doctor	58.28	55.56	0.93	55.13	54.02	0.66	65.81	39.68	**0.0008**	48.15	58.82	0.54	58.58	41.18	0.14
	head of the ward	37.09	27.78	0.73	35.90	31.00	0.44	44.44	19.05	**0.001**	25.93	36.60	0.45	36.09	23.53	0.48
	nurse	25.34	22.22	0.61	26.32	20.93	0.71	27.43	17.74	**0.05**	22.22	24.32	0.94	25.61	11.76	0.34
	patient's family	50.00	55.56	0.79	48.72	50.00	0.92	48.28	49.21	0.35	60.71	46.36	0.34	50.60	29.41	**0.05**
	court	18.79	27.78	0.10	23.08	12.64	0.19	23.48	11.11	**0.02**	7.41	21.19	0.20	18.56	17.65	0.68
Decision to limit futile therapy would be facilitated by	unambiguous legal act	76.13	83.33	0.55	80.77	72.22	0.38	79.13	62.50	**0.05**	70.00	78.57	0.58	76.88	76.47	0.73
	precise selection criteria	84.52	88.89	0.81	88.46	81.11	0.34	80.00	71.88	0.38	83.33	86.36	0.86	86.71	70.47	0.45
	education	74.19	77.78	0.72	76.92	70.00	0.58	89.17	81.25	**0.08**	73.33	74.68	0.98	74.57	70.59	0.37
	declaration of will	80.39	77.78	0.58	79.22	77.53	0.88	78.33	67.19	0.25	72.41	81.7	0.18	80.12	70.59	0.55

ICU – Intensive Care Unit, F – female, M – male, p – statistical significance coefficient, L – lower education, H – higher education

Supplementary Table S3. Differentiation of the respondents' answers to the most important questions of the questionnaire depending on gender, along with the significance of the chi-squared test.

Questionnaire question	% of affirmative responses		Significance of the chi-squared test
	Female	Male	p
Sufficient preparation to talk about futile therapy	6.95	16.67	0.21
Use of futile therapy is a mistake	31.61	33.33	0.10
Decision to limit futile therapy in adults should be made by			
patient	83.23	94.44	0.43
doctor	67.10	50.0	0.31
head of the ward	49.03	38.89	0.71
patient's family	50.32	55.56	0.38
court	28.48	38.89	0.64
Decision to limit futile therapy in children should be made by			
doctor	65.81	61.11	0.91
head of the ward	43.87	50.00	0.77
patient's family	29.41	44.44	0.42
court	65.81	66.67	0.99
Idea of limiting futile therapy is right	64.94	66.67	0.94
Comfort of the patient's last days of life is more important than futile therapy	90.32	94.44	0.56
Decision to limit futile therapy would be facilitated by			
unambiguous legal act	76.13	83.33	0.55
precise eligibility criteria for limiting therapy	84.52	88.89	0.81
education in this field	74.19	77.78	0.72
patient's declaration of will/ living will	80.39	77.78	0.58
Permission to limit futile therapy in relation to oneself	75.66	62.5	0.20
Who would decide to limit futile therapy in relation to the subject			
subject in a declaration of will	88.89	94.44	0.75
doctor	58.28	55.56	0.93
head of the ward	37.09	27.78	0.73
nurse	25.34	22.22	0.61
patient's family	50.00	55.56	0.79
court	18.79	27.78	0.10
